# Single-cell Genomic Analysis of Fiber-adhering Rumen Bacteria during an *in situ* Incubation of Timothy Hay

**DOI:** 10.1264/jsme2.ME26040

**Published:** 2026-09-29

**Authors:** Shuhei Takizawa, Miho Fujimori, Takumi Shinkai

**Affiliations:** 1 Institute of Livestock and Grassland Science, National Agriculture and Food Research Organization, 2 Ikenodai, Tsukuba, Ibaraki, Japan

**Keywords:** carbohydrate-active enzyme, cattle, microbial community, rumen, single-cell genome

## Abstract

Rumen microorganisms adhere to the surface of ingested feed and degrade fibrous compounds into short-chain fatty acids, which are the primary source of energy for milk and meat production. A more detailed understanding of uncultured ruminal bacteria involved in polysaccharide degradation may help improve the feed digestion efficiency of ruminants. The aim of the present study was to identify the ability of uncultured ruminal bacteria to degrade polysaccharides by constructing bacterial genomes and linking their 16S rRNA genes with functional genes using single-cell genome sequencing. The ruminal fibrolytic community was enriched on the surface of Timothy hay via a time-limited *in situ* incubation. A single-cell genomic anal­ysis was used to construct 83 single-cell-assembled genomes from the ruminal fibrolytic community, and 16S rRNA genes were recovered from 80 genomes. The uncultured *Ruminococcus*, *Ruminococcus_D*, and *Fibrobacter* groups had a high percentage of carbohydrate-binding module genes involved in attachment to fibrous compounds. Uncultured *Prevotella* showed different profiles of genes encoding major carbohydrate-active enzymes from those of known *P. ruminicola* spp., and the amino acid sequence of glycoside hydrolase family 43 had various deletions and substitutions. The single-cell genomic anal­ysis enabled the construction of genomes with 16S rRNA genes from fiber-adherent rumen bacteria and linked their 16S rRNA genes with the DNA and amino acid sequences of functional genes involved in carbohydrate utilization. Several uncultured rumen bacteria possess functional genes that contribute to feed digestion and biofilm formation.

The rumen of cows contains a complex microbial ecosystem comprising various bacteria, archaea, protozoa, and fungi. Rumen microorganisms adhere to the surface of ingested feed and secrete fibrolytic enzymes that hydrolytically degrade fibrous compounds into oligosaccharides and monosaccharides. The saccharides produced are converted into short-chain fatty acids (SCFAs), predominantly acetate, propionate, and butyrate, which are absorbed through the rumen epithelium and utilized by the host as a primary source of energy for milk and meat production. Therefore, ruminal microorganisms affect feed efficiency and milk production (Cammack *et al.*, 2018). A more detailed understanding of the fiber digestion system of ruminal microorganisms may help improve feed digestion and energy utilization in cows.

Hungate (1969) developed a strictly anaerobic culture method that enabled the cultivation of rumen bacteria and investigated their functions in the rumen (Creevey *et al.*, 2014; Nyonyo *et al.*, 2014). However, less than 15% of ruminal bacteria may be cultured using anaerobic culture methods (Edwards *et al.*, 2004; Morgavi *et al.*, 2013). Culture-independent techniques are widely used to investigate the genes and genomes of uncultured rumen bacteria involved in feed digestion (Stewart *et al.*, 2019; Takizawa *et al.*, 2023). Amplicon sequencing is a targeted approach for analyzing genetic variations in specific genes, particularly the 16S and 18S rRNA genes and the ITS region. This targeted approach may reveal the taxonomic composition of‍ ‍the rumen microbial community, but cannot predict microbial metabolic functions (Denman *et al.*, 2018). Metagenomic sequencing is an untargeted approach for comprehensively analyzing all genes of an entire microbial community. This untargeted approach may be used to construct draft genomes of uncultured microorganisms and profile not only the taxonomic composition, but also functional potential. Previous metagenomic studies identified functional genes and revealed the profile and number of carbohydrate-active enzymes (CAZymes) in the fiber-adherent rumen microbial community, providing important information for elucidating the mechanisms underlying fiber digestion in the rumen (Brulc *et al.*, 2009; Wang *et al.*, 2013). However, 16S rRNA genes, as a phylogenetic marker, are often not found in metagenome-assembled genomes (Stewart *et al.*, 2019) because assembly algorithms for metagenomic data fail to resolve the highly conserved sequence regions of 16S rRNA genes from related bacteria (Song *et al.*, 2022). Single-cell amplified genomes (SAG) in gel bead sequencing have been developed to overcome this issue associated with metagenomic sequencing. This parallel sequencing technique utilizes a gel matrix that enables serial enzymatic reactions for small genome amplifications and provides support for the isolation of amplified genomes (Chijiiwa *et al.*, 2020). The SAG genome has shown a high recovery rate of 16S rRNA genes, comparative genomics at the strain level, and the detection of characteristic genes in soil, seawater, and gut microbial communities (Chijiiwa *et al.*, 2020; Nishikawa *et al.*, 2022). The rumen microbiomes of beef and draft cattle have also been investigated using high-throughput single-cell genome sequencing to compare bacterial compositions and metabolic pathways between breeds (Guan *et al.*, 2025). However, 16S rRNA genes and functional genes are not linked in uncultured rumen bacteria involved in feed digestion, and their phylogenetic and functional characteristics remain unclear. Therefore, we hypothesized that a single-cell genomic sequencing technique may be used to construct a genome with the 16S rRNA genes of uncultured rumen bacteria involved in feed digestion, which may reveal their phylogenetic and functional characteristics and provide novel insights into the role of fiber-adherent rumen microorganisms. Therefore, the present study aimed to construct genomes with the 16S rRNA genes of uncultured fiber-adherent rumen microorganisms via single-cell genomic sequencing and link their taxonomy and function, particularly CAZymes, which contribute to fiber digestion.

## Materials and Methods

### Animals and *in situ* incubation

This study was conducted in accordance with the guidelines of the Animal Care Committee of the National Agriculture and Food Research Organization (NARO) (approval no. 21B194ILGS). Three fistulated dry Holstein cows maintained at NARO were used for an *in situ* incubation. The cows were fed a diet consisting of Timothy hay and concentrate at a ratio of 7:3 (w/w) twice daily (09:30 and 16:30) to exceed their maintenance energy level. Timothy hay was used as the substrate for the *in situ* incubation. Timothy hay contained 91.3% dry matter (DM), 19.2% DM crude protein, 1.7% DM ether extract, 11.9% DM crude ash, 66.2% DM neutral detergent fiber (NDF), 36.4% DM acid detergent fiber (ADF), and 7.1% DM acid detergent lignin (ADL). Timothy hay was ground to pass through a 1.00-mm screen, and 5 g of this ground hay was placed in 5×10-cm polyester bags with a pore size of 53 μm (BG0510; SANSHIN). The bags were heat-sealed, placed inside a mesh bag, and inserted into the rumen via a cannula (three bags per digestion period per cow) at 10:30 h. The *in situ* incubation was performed for 0.5, 24, 48, and 72 h. Three bags were removed from the rumen during each digestion period, washed with sterile saline until the saline became clear, and squeezed to remove the water. The bags were then opened and used to prepare fiber-adherent microbial samples for scanning electron microscopic observations and to assess the digestibility of Timothy hay.

### Detachment of fiber-adherent microorganisms

The detachment of fiber-adherent microorganisms from the surface of Timothy hay was conducted following the method described by Minato and Suto (1978), with some modifications. Samples were mixed with 30 mL of phosphate-buffered saline containing 0.1% methylcellulose and centrifuged at 300×*g* for 5 min to remove fibrous particles. The supernatant containing the detached fiber-adherent microorganisms was collected as a detached microbial suspension. A portion of the microbial suspension was mixed with an equal volume of 30% glycerol and stored at –80°C to suppress microbial activity until a single-cell genomic anal­ysis. The remaining samples were stored at –30°C for DNA extraction.

### Real-time PCR and amplicon sequencing

DNA extraction, real-time PCR, and amplicon sequencing were performed as previously reported (Takizawa *et al.*, 2023). Briefly, total DNA was extracted from 1 mL of the fiber-adherent microbial suspension using a FastDNA SPIN Kit (MP Biomedicals), according to the manufacturer’s instructions. Real-time PCR was conducted following a previously reported method (Takizawa *et al.*, 2023), using primer sets (1114F and 1275R) to amplify the bacterial 16S rRNA gene (Denman and McSweeney, 2006). DNA libraries were constructed using a primer set for the bacterial 16S rRNA V3–V4 region (341F, 5′-CCTACGGGNGGCWGCAG-3′; 805R, 5′-GACTACHVGGGTATCTAATCC-3′) (Herlemann *et al.*, 2011). The constructed libraries were sent to Bioengineering Lab. for 300-bp paired-end sequencing using the Illumina MiSeq platform (Illumina). Read processing, operational taxonomic unit (OTU) clustering with 97% similarity, and annotation to the SILVA database (version 138.99) were performed using QIIME2 plugins (version 2023.4). Rarefaction of the reads and anal­yses of alpha diversity indices (Shannon and Simpson indices) and non-metric multidimensional scaling based on Bray–Curtis dissimilarity were conducted using the R package vegan (version 2.5-7; Oksanen *et al.*, 2022). Sequence data for the amplicon anal­ysis were deposited in the DNA Data Bank of Japan sequence read archive under accession number DRR753613–DRR753624 (DRA022652).

### Single-cell genomic anal­ysis

Single-cell genomes were constructed using microbial suspensions following a 48-h *in situ* incubation, when both microbial adherence and fibrous digestion were observed. Fiber-adherent microbial suspensions were shipped on dry ice to BitBiome. The suspensions were passed through filter membranes with a pore size of 35 μm to remove bovine tissues and cells and were centrifuged at 2,000×*g* for 30 s. The precipitates were suspended with PBS and centrifuged at 8,000×*g* for 5 min. This washing procedure was repeated thrice to wash microbial cells and remove contamination by bovine DNA. A SAG library of the microbial cells in the samples was constructed using the SAG gel method (Chijiiwa *et al.*, 2020; Arikawa *et al.*, 2021; Ide *et al.*, 2022; Nishikawa *et al.*, 2022). In total, 768 gel droplets were sequenced using an Illumina NextSeq2000 System with a 2×150-bp configuration. Adapter sequences and low-quality reads were eliminated from raw sequence reads of single-cell genome sequences using bbduk.sh (version 38.90) with the following parameters: (ktrim=r ref=adapters k=23‍ ‍mink=11 hdist=1 tpe tbo qtrim=r trimq=10‍ ‍minlength=40 maxns=1‍ ‍minavgquality=15). The reads mapped to the masked human genome were eliminated using bbmap.sh (version 38.90) for removing human DNA with the following parameters: (quickmatch fast untrim minid=0.95 maxindel=3 bwr=0.16 bw=12 minhits=2, path=human_masked_index qtrim=rl trimq=10). Data on the human masked index, hg19_main_mask_ribo_animal_allplant_allfungus.fa.gz, were obtained using previously published data (https://zenodo.org/record/1208052#.X1hBFWf7SdY). These quality-controlled reads of single-cell genomes were assembled *de novo* into contigs using SPAdes (v3.15.2; Bankevich *et al.*, 2012) with the following parameters: (--sc --careful --disable-rr --‍disable-gzip-output –k 21,33,55,77,99,127). Contigs shorter than 200 bp were excluded from the SAG assemblies. Coding DNA sequences (CDSs), rRNAs, and tRNAs were predicted from SAGs using Prokka (v1.14.5; Seemann, 2014) with the following parameters: (--raw product --mincontiglen 200). The quality of the contigs was evaluated using QUAST (v5.0.2) with the default options. The completeness and contamination of SAGs were evaluated using CheckM (v1.1.3; Parks *et al.*, 2015) in a lineage workflow with the parameters (-r --nt) or in a taxonomy workflow with the parameter (--nt domain bacteria). To exclude low-quality SAGs, the cut-off value of completeness was set at 50% and that of contamination at 10% (Ide *et al.*, 2022). After quality control, 83 SAGs remained with 48×10^6^ reads (average 579×10^3^ reads per SAG) and 7.3×10^9^ bases (average 87.5×10^6^ bases per SAG). Taxonomic identification was performed using Genome Taxonomy Database (GTDB)-Tk (version 2.3.2; Chaumeil *et al.*, 2022) with the default options and GTDB Release 214. The novelty score of SAGs was calculated from average nucleotide identity and alignment fraction (Varghese *et al.*, 2015). A phylogenetic tree of bacterial SAGs was constructed using bcgTree (v1.2.0; Ankenbrand and Keller, 2016) with the default options and visualized using the Interactive Tree of Life (https://itol.embl.de). The 16S rRNA genes were recovered from SAGs and searched using BLAST against the NCBI rRNA/ITS databases to calculate identity with known species. SAGs were submitted to a local version of dbCAN2 (Zhang *et al.*, 2018) to detect genes encoding CAZymes. The number of CAZyme genes was normalized by the number of CDSs. The R package gplots (version 3.2.0; Warnes *et al.*, 2024) was used to calculate and construct a heatmap of CAZymes. Sequence data for the single-cell genomic anal­ysis were deposited in the DNA Data Bank of Japan sequence read archive under the accession number DRR753934–DRR754016 (DRA022660).

### Scanning electron microscopic observations

The stems and leaves of Timothy hay were cut into 2-mm squares. These pieces were pre-fixed with 2% paraformaldehyde and 2% glutaraldehyde in 0.1 mol L^–1^ cacodylate buffer (pH 7.4) at 4°C overnight, fixed with 1% tannic acid in 0.1 mol L^–1^ cacodylate buffer at 4°C for 2 h, washed four times with 0.1 mol L^–1^ cacodylate buffer at 4°C for 30 min, and then post-fixed with 2% osmic acid in 0.1 mol L^–1^ cacodylate buffer at 4°C for 3 h. The samples were then dehydrated using a graded ethanol series (50%, 70%, 90%, and absolute ethanol), followed by overnight soaking in absolute ethanol. The dehydrated samples were then soaked in a mixture of absolute ethanol and t-butanol (1:1) for 1 h, soaked thrice in t-butanol for 30 min, and then freeze-dried. The samples were then coated with osmium using an Osmium Plasma Coater (NL-OPC80NS; Nippon Lase & Electronics Laboratory) and observed under a scanning electron microscope (SEM) (JSM-7500F; JEOL).

### Chemical and statistical anal­yses

Samples were analyzed for DM, NDF, ADF, and ADL contents (Association of Official Analytical Chemists, 1990; Van Soest *et al.*, 1991; Hiraoka *et al.*, 2012). The disappearance of these components during the *in situ* incubation was calculated as follows: Disappearance (%)=100–(weight of components after incubation [g]/initial weight of components [g])×100.

Statistical anal­yses were performed using JMP software (version 17.2, SAS Institute) and R version 4.1.1. Statistical differences in digestibility, alpha diversity, and bacterial abundance were analyzed using the Wilcoxon rank-sum test with the Bonferroni adjustment. The significance of clustering for NMDS was analyzed using a permutational multivariate anal­ysis of variance with the adonis2 function in the vegan package. The significance of differences was set at *P*<0.05.

## Results

### Fiber digestion and adherence of rumen microorganisms

The DM, NDF, and ADF components were temporally degraded during the *in situ* incubation, particularly between 0.5 and 24 h; ADL degradation was observed after 24 h (Fig. 1). The disappearance rates of DM, NDF, ADF, and ADL after 72 h were 61.3, 55.0, 54.0, and 24.6%, respectively.

Real-time PCR using the bacterial 16S rRNA gene primers showed that the abundance of fiber-adhering bacteria increased 21.3-fold between 0.5 and 24 h (Fig. 2A). According to the amplicon sequencing anal­ysis of the bacterial 16S rRNA V3–V4 region, there was no significant difference in the Simpson index during the *in situ* incubation. The Shannon index significantly decreased between 0.5 and 24 h, but slightly increased at 48 h (Fig. 2B). Non-metric multidimensional scaling indicated that the structure of the bacterial community changed throughout the *in situ* incubation period (Fig. 2C). In total, 31 phyla were detected in the amplicon sequencing anal­ysis during rumen digestion. *Bacillota* was the dominant phylum, followed by *Bacteroidota* (Fig. 2D). The relative abundance of both *Fibrobacterota* and *Spirochaetota* increased during the first 24 h, whereas those of *Bacteroidetes* and *Verrucomicrobiota* decreased during the same period. Morphological observations via scanning electron microscopy revealed that various rumen microorganisms adhered to the hay surface after 24 h, after which the fibrous structure was broken (Fig. 3).

### Quality and taxonomy of constructed genomes

We selected samples obtained after 48 h of the *in situ* incubation for the single-cell genomic anal­ysis because rumen microorganisms were enriched on the surface of‍ ‍Timothy hay. We obtained 83 quality-controlled SAGs from the 768 gel droplets sequenced. The completeness, contamination, total length, and N50 of the 83 SAGs were‍ ‍62.97±11.27%, 4.41±2.17%, 2.36±0.60 Mb, and 15.18±10.94‍ ‍kb, respectively (Fig. 4, Table S1). The 16S rRNA genes were recovered from 80 SAGs (96.4% of total SAGs), 67 of which were longer than 1,000 bp (Fig. 4). Seven prokaryotic phyla, including one archaeal phylum, were detected using the single-cell genomic anal­ysis (Fig. 5). *Bacillota* (38/83 SAGs) was the most dominant phylum, followed by *Bacteroidota* (25/83 SAGs). Based on GTDB, 78 SAGs were classified at the genus level (Table S2), where *Prevotella* (14/83 SAGs) was the most dominant, followed by *Fibrobacter* (10/83 SAGs) and *Ruminococcus* (9/83 SAGs). Four SAGs were identified at the species level as three cultured rumen bacteria (*Prevotella ruminicola*, *Ruminococcus albus*, and *Streptococcus infantarius*); the remaining 79 SAGs were either not identified at the species level or were identified as the constructed genomes of uncultured bacteria. In terms of taxonomic novelty, 61 SAGs showed a novelty score of more than 50% based on GTDB, and 62 SAGs exhibited less than 97% identity to the reference 16S rRNA gene sequence of the cultured bacteria.

### Resolution of the single-cell genomic anal­ysis for rumen microorganisms

The gene profiles of CAZymes in the 83 SAGs were compared (Fig. 6A, Table S3). The profiles of CAZyme genes include auxiliary activity (AA), carbohydrate-binding module (CBM), carbohydrate esterase (CE), cohesion, glycoside hydrolase (GH), glycosyltransferase (GT), and polysaccharide lyase (PL). The number and composition of CAZyme genes differed at the phylum and genus levels. *Prevotella*, *Fibrobacter*, *Ruminococcus*, *Ruminococcus*_D, and *Butyrivibrio* had CAZyme gene percentages of >5%, higher than those of other bacteria. In terms of CAZyme gene compositions, three genera, *Ruminococcus*, *Ruminococcus_D*, and *Fibrobacter*, exhibited a high percentage of CBM genes, whereas *Prevotella* had a high percentage of GH genes. Since the CAZyme gene profiles differed among the SAGs of the dominant genus *Prevotella*, these SAGs were further compared to clarify the genomic differences (Fig. 6B). The CAZyme profile of *P. ruminicola* (48h3_00106, 99.3% identity of 16S rRNA) was different from those of other uncultured *Prevotella* spp. (identity <95%). Three SAGs (48h1-00195, 48h1-00294, and 48h3-00014) showed more than 95% identity of 16S rRNA genes with each other and were closely related in the phylogenetic tree; therefore, these SAGs differed at the species or strain levels. The profiles of the major CAZyme genes differed among these three SAGs (Fig. 6B). A comparison of the amino acid sequences of GH43 showed some deletions and substitutions (Fig. 6C).

### Identification of key players in fiber adherence and digestion

Thirty-seven CBM family genes were detected in the 83 SAGs. The dominant CBM families, CBM6 (average 0.13% of CDSs in 83 SAGs), CBM22 (0.08%), CBM4 (0.06%), CBM35 (0.04%), and CBM37 (0.04%), accounted for 62% of all CBM genes (Table S3). Three SAG groups, including cultured and uncultured *Ruminococcus*, *Ruminococcus_D*, and *Fibrobacter*, possessed 1.26, 2.48, and 0.89% of CBM genes, respectively (Fig. 7A), which were significantly higher than those of the other SAGs (average, 0.08%). The composition of CBMs differed among *Ruminococcus*, *Ruminococcus_D*, and *Fibrobacter*. The CBM profiles of the *Ruminococcus*, *Ruminococcus_D*, and *Fibrobacter* groups were distinctly characterized by high percentages of CBM22 (0.60% of CDSs in the *Ruminococcus* group), CBM37 (1.83% in the *Ruminococcus_D* group), and CBM6 (0.64% in the *Fibrobacter* group), respectively. Ninety-five GH families (average 2.54% of CDSs in 83 SAGs) were detected in the 83 SAGs, with GH43 showing the highest percentage (0.30%), followed by GH5 (0.17%), GH3 (0.16%), GH13 (0.15%), and GH10 (0.12%) (Table S3). The *Ruminococcus*, *Ruminococcus_D*, and *Fibrobacter* groups exhibited high percentages of GH genes (3.44, 2.43, and 2.74% of CDSs, respectively), as well as CBM; however, the profile of GH genes significantly differed among these groups (Fig. 7B). The GH profile of the *Ruminococcus* group was characterized by GH11 (0.63% of CDSs in the *Ruminococcus* group), whereas that of the *Ruminococcus_D* group was characterized by GH5 (0.42% in the *Ruminococcus_D* group). The *Fibrobacter* group was characterized by GH5 (0.35% in the *Fibrobacter* group) and GH43 (0.33%). Other genera, such as *Saccharofermentans*, *Streptococcus*, and *Treponema*, showed a lower percentage of CBM and GH genes than the *Ruminococcus*, *Ruminococcus_D*, and *Fibrobacter* groups (Table S3).

### Rumen bacteria involved in biofilm formation and lignin digestion

The SAGs of the *Prevotella* group had 0.11% of CBM genes (Fig. 7A); however, some had more than 4% GH genes of CDSs (Fig. 7B). Some SAGs from *Butyrivibrio*, *Blautia_A*, and uncultured *Bacterodaceae* also contained a high percentage of GH genes (Fig. 7B). Two GT families (GT2 and GT4), which are involved in biofilm formation (Ma *et al.*, 2025), were found in all SAGs, accounting for 73.0% of total GT. *Prevotella*, *Butyrivibrio*, *Blautia_A*, and uncultured *Bacteroidaceae* showed a higher percentage of GT genes than CBM genes (Fig. 7A). The AA4 gene encoding vanillyl-alcohol oxidase, converting a wide range of phenolic compounds bearing side chains at the para-position of the aromatic ring, was detected in two SAGs of *Succiniclasticum* (48h1-00183) and *Christensenellales_RUG563* (48h1-00011) (Table S3). Cytochrome c551 peroxidase (EC 1.11.1.5), which is involved in the oxidation of lignin and aromatic compounds (Silva *et al.*, 2021), was detected in three SAGs of uncultured *Bacteroidales_F082* (48h1-00206 and 48h1-00296) and *WCHB1_69* (48h1-00123) (Table S3).

## Discussion

*In situ* techniques using nylon bags are suitable for kinetic studies following the time course of the disappearance of feedstuff, and they have been widely used to evaluate the rate and extent of digestion in the rumen (Ørskov, 2000; López, 2005). We adopted this technique to investigate the disappearance of Timothy hay, which enabled the time at which polysaccharide components were effectively digested to be identified. The real-time PCR anal­ysis revealed that rumen bacteria adhered to the hay surface immediately after the start of the *in situ* incubation and digested non-fibrous components—consistent with surface morphology observations. NDF and ADF disappeared throughout the *in situ* incubation, whereas ADL was digested from 24 to 48 h (Fig. 1), indicating that the digested components dynamically changed during the *in situ* incubation process. This result is consistent with the findings of previous studies on ADL digestion during an *in situ* incubation (Gharechahi *et al.*, 2022; Firrincieli *et al.*, 2024), which suggested that lignin is a physical barrier for rumen microorganisms and their enzymes (Gharechahi *et al.*, 2022). Similar to the dynamics of fiber digestion, the structure of fiber-adhering rumen microorganisms dynamically changed throughout the *in situ* incubation, and bacterial diversity generally increased from 24 to 48 h (Fig. 2). Microscopy enabled the morphology of fiber-adherent rumen bacteria to be visualized and revealed changes in bacterial diversity on the surface of Timothy hay during the *in situ* incubation (Fig. 3). Based on these results, we selected the samples obtained after 48 h of the *in situ* incubation for the single-cell genomic anal­ysis because rumen microorganisms involved in polysaccharide deconstruction were enriched on the surface of Timothy hay.

The single-cell genomic anal­ysis in the present study yielded 83 SAGs, including genomes of uncultured bacteria from 768 gel droplets (Fig. 5). This low yield may be attributed to sample processing, particularly freezing and the single-cell suspension, for the single-cell genomic anal­ysis. Chijiiwa *et al.* (2020) performed a single-cell genomic anal­ysis of the mouse gut microbiota from fecal samples stored at –80°C and obtained 44 high- and medium-quality SAGs from 464 droplets. It was assumed that bacterial cells were damaged during the freezing process, which resulted in a failure in cell lysis, genome amplification, and/or sequencing. Our microscopic observations showed that rumen bacteria aggregated with other microorganisms and/or formed biofilms, indicating that the aggregate and biofilm of fiber-adherent bacteria contaminated some cells in a droplet and increased the percentage of low-quality SAGs. The optimization of sample processing is required to obtain more high-quality SAGs from fiber-adherent rumen bacteria. Despite the low yield of SAGs, the single-cell genomic anal­ysis resulted in the high recovery rate of 16S rRNA genes in SAGs (Fig. 4). A single-cell genomic anal­ysis has been used to obtain a massive number of microbial genomes from environmental samples, and has enabled the detection of specific genes (Kogawa *et al.*, 2018; Nishikawa *et al.*, 2022). The metagenome sequencing of rumen bacteria was previously used to construct 4,941 metagenome-assembled genomes; however, 4,477 of them (90.6%) did not contain 16S rRNA genes (Stewart *et al.*, 2019). This low recovery rate of 16S rRNA genes was due to the highly conserved sequence regions that often caused misassembly. The present results showed that the single-cell genomic anal­ysis enabled the construction of genomes with a high recovery rate of 16S rRNA genes from fiber-adherent rumen bacteria and the linkage of their 16S rRNA genes with functional genes.

To identify microbial adherence and feed digestion potential, CAZyme gene profiles were compared among the SAGs. CBM is a protein that binds polysaccharides and plays an important role in the adherence of ruminal bacteria. Our single-cell genomic anal­ysis showed that the SAGs of the uncultured *Ruminococcus*, *Ruminococcus_D*, and *Fibrobacter* groups possessed a higher percentage of CBM genes than other rumen bacteria (Fig. 7A). Jose *et al.* (2017) investigated the CAZyme gene of a rumen microbial community using a metagenomic anal­ysis and found that a major portion of CBM genes corresponded to the genera *Ruminococcus*, *Fibrobacter*, and *Prevotella*. Our anal­ysis also showed that the CBM profiles of the *Ruminococcus*, *Ruminococcus_D*, and *Fibrobacter* groups were characterized by high percentages of CBM22, CBM37, and CBM6, respectively. CBM22, CBM37, and CBM6 are specifically detected in *R. flavefaciens*, *R. albus*, and *Fibrobacter succinogenes* and bind to various polysaccharides, xylan, and single-chain hemicellulose, respectively (Ezer *et al.*, 2008; Berg Miller *et al.*, 2009; Suen *et al.*, 2011). In addition, the GH profiles of the uncultured *Ruminococcus*, *Ruminococcus_D*, and *Fibrobacter* groups were characterized by high percentages of GH11, GH5, and GH43 (Fig. 7B). GH5 exhibits a broad substrate specificity, while GH11 and GH43 are responsible for xylanase activities. These results suggest that the uncultured *Ruminococcus*, *Ruminococcus*_D, and *Fibrobacter* groups possess various CBMs for adhering to the surface of Timothy hay, contributing to the digestion of polysaccharides by GHs with various enzyme activities. In contrast, *Saccharofermentans*, *Streptococcus*, and *Treponema* showed lower percentages of‍ ‍CBM and GH genes than the *Ruminococcus*, *Ruminococcus_D*, and *Fibrobacter* groups. These genera cannot hydrolyze fibrous cellulose or xylan in the rumen, but utilize starch, oligosaccharides, and monosaccharides (Stanton and Canale-Parola, 1980; Chen *et al.*, 2010; Hua *et al.*, 2022). The present study used Timothy hay mainly composed of fibrous compounds, suggesting that *Saccharofermentans*, *Streptococcus*, and *Treponema* utilize oligosaccharides and monosaccharides produced by other fibrolytic bacteria in the biofilm on the surface of Timothy hay.

The microbial colonization of feed particles is fundamental to fiber digestion; rumen microbes that form tight biofilms are key players in this process (Gharechahi *et al.*, 2022). The *Prevotella*, *Butyrivibrio*, *Blautia_A*, and uncultured *Bacteroidaceae* groups exhibited a high ratio of GTs involved in biofilm formation (Fig. 7A). Several species of *Prevotella* and *Butyrivibrio* are known to produce exopolysaccharides involved in biofilm formation (Kelly *et al.*, 2010; Yamanaka *et al.*, 2011; Albaghdadi *et al.*, 2021), indicating that the *Prevotella* and *Butyrivibrio* groups produce exopolysaccharides to form biofilms on the surface of Timothy hay and enable the colonization of the rumen microbial community. However, the functions of biofilm formation and feed digestion in the *Blautia_A* and uncultured *Bacteroidaceae* groups remain unclear. Therefore, further research is required to identify the roles of these bacteria in biofilm formation and feed digestion.

The functional genes encoding AA4 and cytochrome c551 peroxidase were detected from SAGs of the *Succiniclasticum*, *Christensenellales_RUG563*, *Bacteroidales F082*, and *WCHB1_69* groups. Several isolates with lignin-degrading activity, such as *Ochrobactrum pseudintermedium*, *Klebsiella pneumoniae*, and *Bacillus sonorensis*, have been found in the rumen (Wang *et al.*, 2021). Lignin is the main plant cell wall component responsible for recalcitrance, and its effects on the indigestibility of NDF are stronger at lower lignin concentrations (Traxler‍ ‍*et al.*, 1998). The present results indicate that the *Succiniclasticum*, *Christensenellales_RUG563*, *Bacteroidales F082*, and *WCHB1_69* groups have functional genes involved in lignin degradation. However, *in vitro* and *in vivo* investigations are needed to confirm their lignin-degrading ability in the ruminal environment.

Genomic information from a metagenomic anal­ysis based on short-/long-read sequencing has been widely used to identify the functional genes of rumen microorganisms. Functional enzymes have been identified from metagenomic data and expressed in various host organisms for agricultural and industrial applications (Ariaeenejad *et al.*, 2024; Tang *et al.*, 2024). The accumulation of metagenomic data of high quality and resolution may be effective for identifying novel and functional proteins from the rumen microbial community. A single-cell genomic anal­ysis may be used to construct the draft genome of rumen microorganisms without misassembly errors, enabling the visualization of small differences in CAZyme profiles and amino acid sequences among SAGs. Our comparative anal­ysis of uncultured *Prevotella* spp. revealed that their amino acid sequences of GH43 (xylanase) showed various deletions and substitutions (Fig. 6). Mutations in amino acid sequences change the structure and function of enzymes (Cheng *et al.*, 2012), suggesting that the function of GH43 was different between the uncultured *Prevotella* spp. and contributed to the digestion of Timothy hay with a complex structure and fibrous polymers. Although further research is required to confirm the accuracy of this anal­ysis and the enzyme activity of GH43 with various deletions and substitutions, a single-cell genome anal­ysis has the potential to identify novel and functional proteins from uncultured rumen bacteria.

In conclusion, a single-cell genomic anal­ysis was used to construct medium- and high-quality genomes with 16S rRNA genes from uncultured rumen bacteria, enabling their 16S rRNA genes to be linked with the DNA and amino acid sequences of functional genes. The present study revealed uncultured rumen bacteria possessing high percentages of CBM and GH genes and identified them as the uncultured *Ruminococcus*, *Ruminococcus_D*, and *Fibrobacter* groups by 16S rRNA gene and genomic classifications. Uncultured *Prevotella* spp. showed different gene profiles encoding major CAZymes from those of cultured *P. ruminicola*, and their amino acid sequences had various deletions and substitutions. The present results suggest that several uncultured bacterial groups contribute to the colonization of the rumen microbial community and polysaccharide digestion. Although further experiments are required to validate and accumulate single-cell genomic data, this study suggests that a single-cell genomic anal­ysis is an effective method to construct genomes with the 16S rRNA genes of uncultured bacteria and link their amino acid sequences to functional genes for mining uncultured, but functional rumen bacteria and their key fibrolytic enzymes.

### Availability of data and materials

The datasets supporting the conclusions of this article are included within the article and Supplementary Material. The authors confirm that all supporting data, code, and protocols have been provided within the article or the supplementary data files.

## Citation

Takizawa, S., Fujimori, M., and Shinkai, T. (2026) Single-cell Genomic Analysis of Fiber-adhering Rumen Bacteria during an *in situ* Incubation of Timothy Hay. *Microbes Environ ***41**: ME26040.

https://doi.org/10.1264/jsme2.ME26040

## Supplementary Material

Supplementary Material 1

Supplementary Material 2

Supplementary Material 3

Supplementary Material 4

## Figures and Tables

**Fig. 1.**
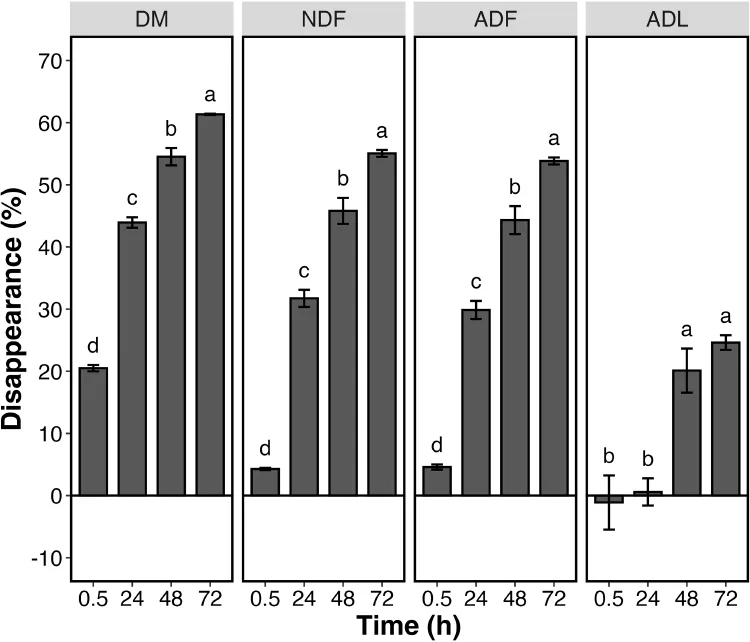
Disappearance of Timothy hay during an *in situ* incubation. Data show the mean of triplicate experiments. Error bars represent the standard error. Different letters indicate significant differences among the incubation times by component (*P*<0.05). DM, dry matter; NDF, neutral detergent fiber; ADF, acid detergent fiber; ADL, acid detergent lignin.

**Fig. 2.**
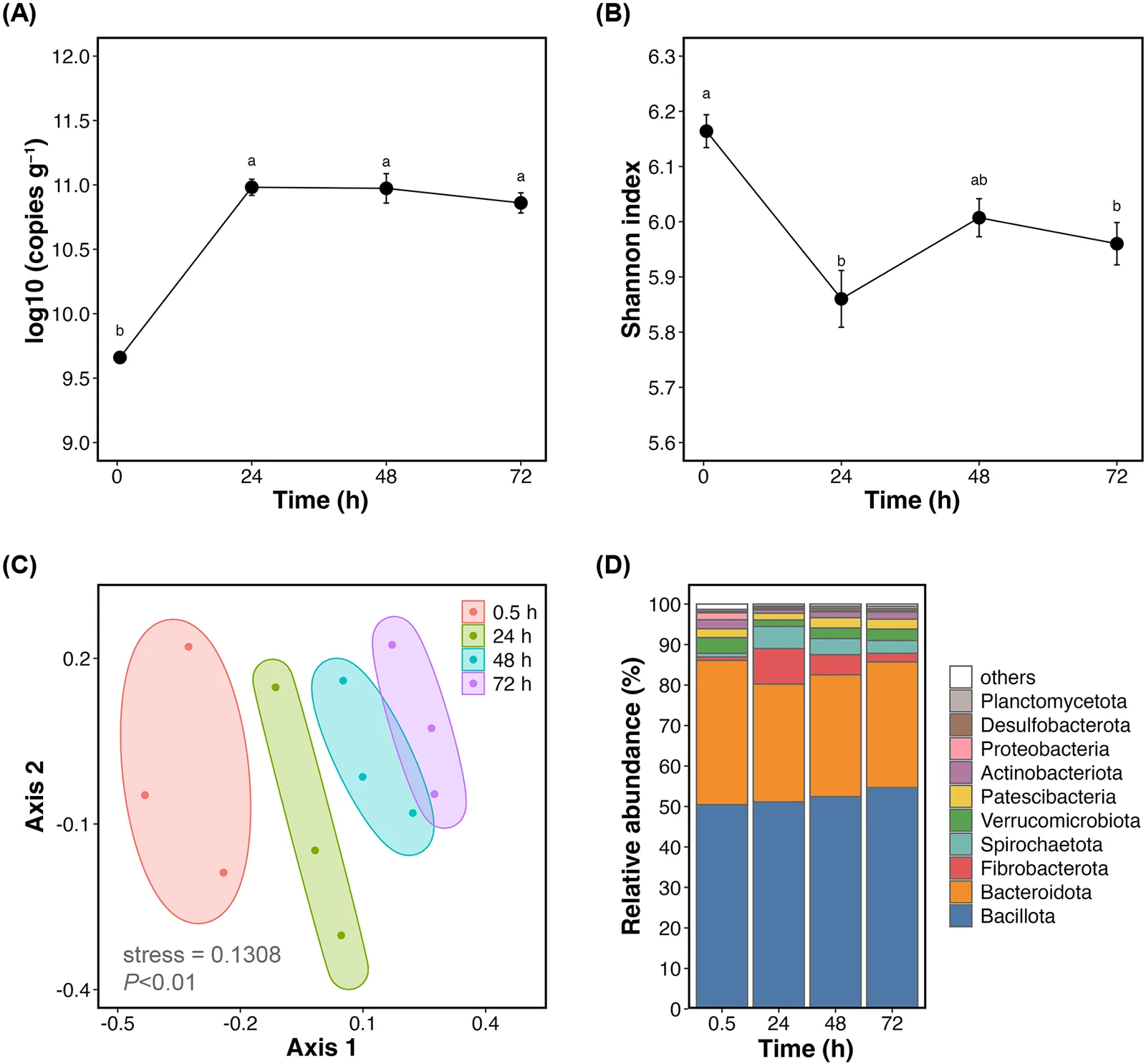
Time-course changes in the fiber-adherent bacterial community that developed on *in situ*-incubated Timothy hay. (A) Absolute abundance of fiber-adherent bacteria, (B) Shannon index, (C) non-metric multidimensional scaling, and (D) taxonomic composition of the fiber-adherent bacterial community at the phylum level. The top 10 phyla are shown, and other phyla are included in “others”. Different letters indicate a significant difference between digestion times (*P*<0.05).

**Fig. 3.**
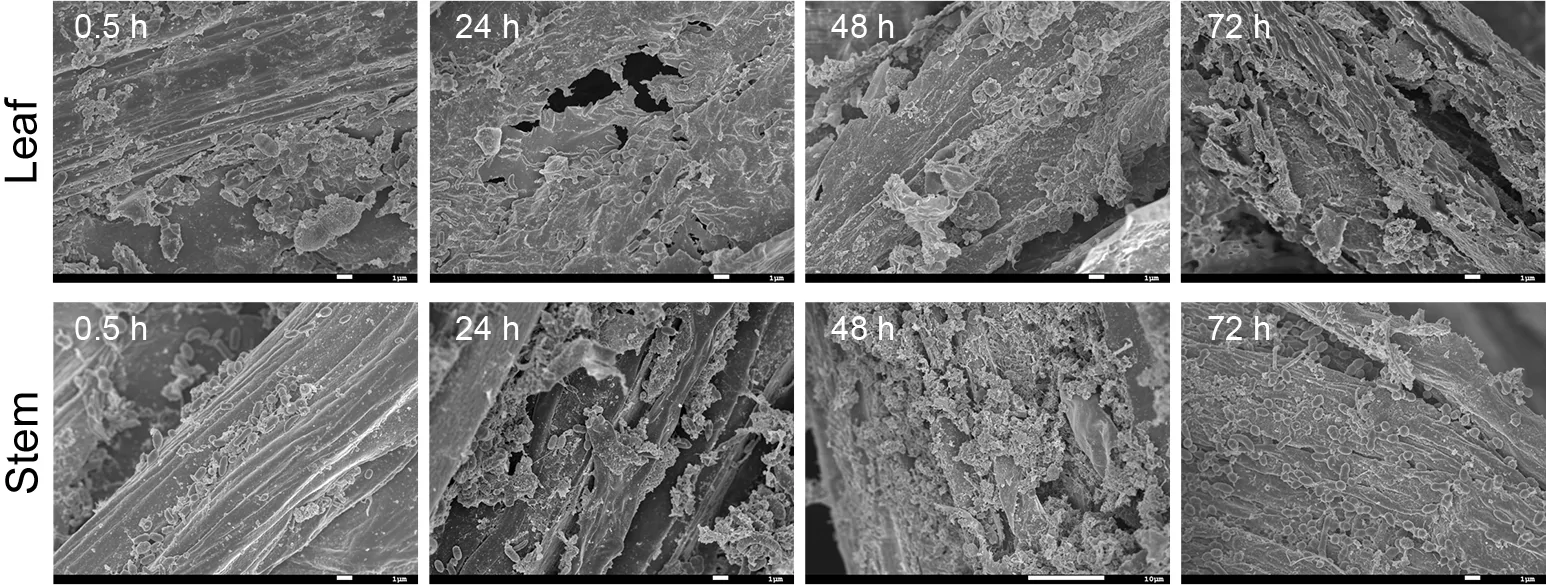
Surface morphologies of the leaf (upper) and stem (lower) of Timothy hay during an *in situ* incubation, as exami­ned using scanning electron microscopy. From left to right: 0.5, 24, 48, and 72 h of *in situ* incubation.

**Fig. 4.**
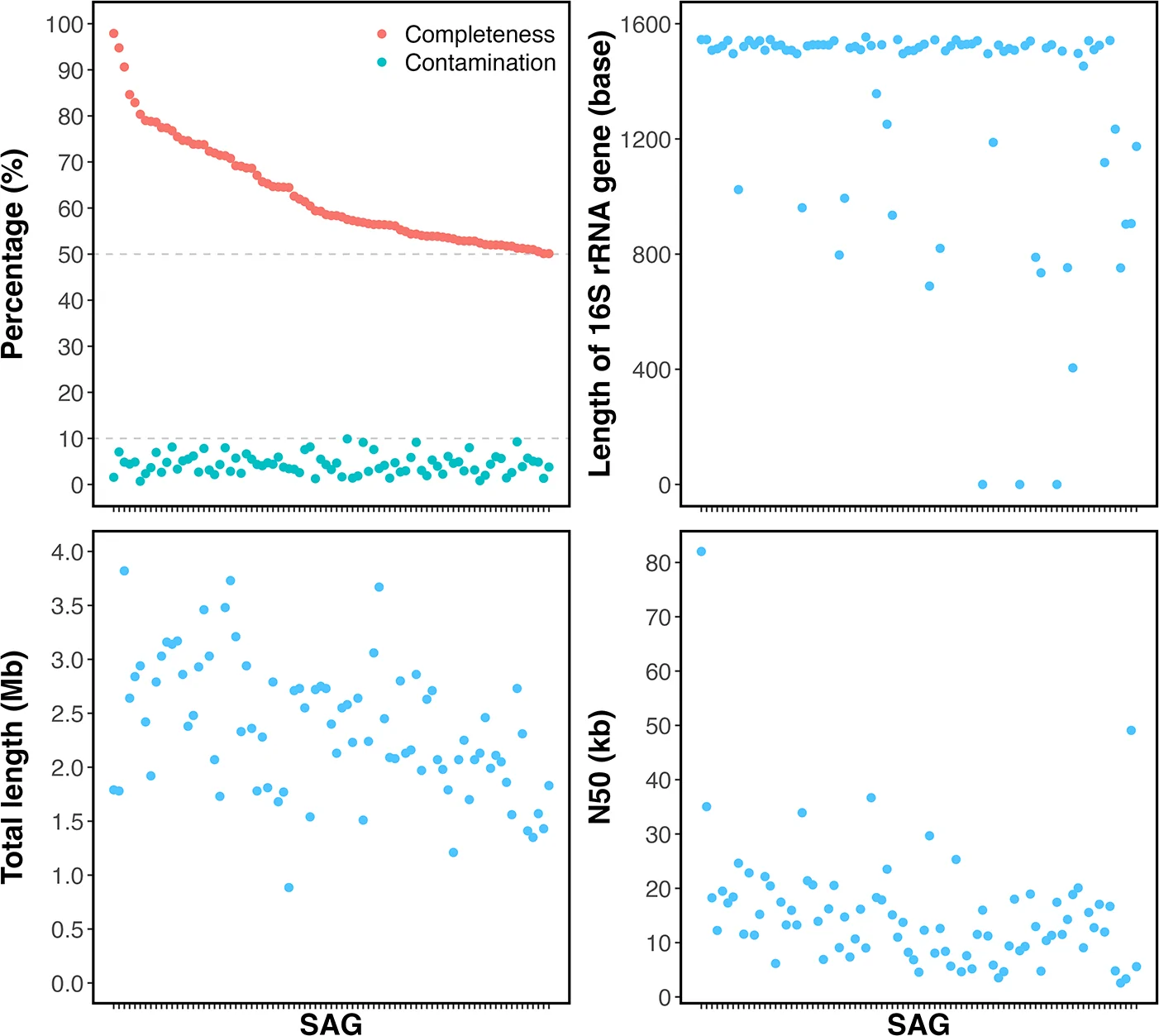
Quality of single-cell amplified genomes (SAGs) of fiber-adherent rumen microorganisms. (Upper left) Completeness and contamination. The dashed lines represent a threshold for 50% of completeness and 10% of contamination. (Upper right) Maximum length of the 16S rRNA gene recovered from SAGs. (Lower left) Total length of the assembly. (Lower right) N50. SAGs are numbered in the order of completeness from left to right.

**Fig. 5.**
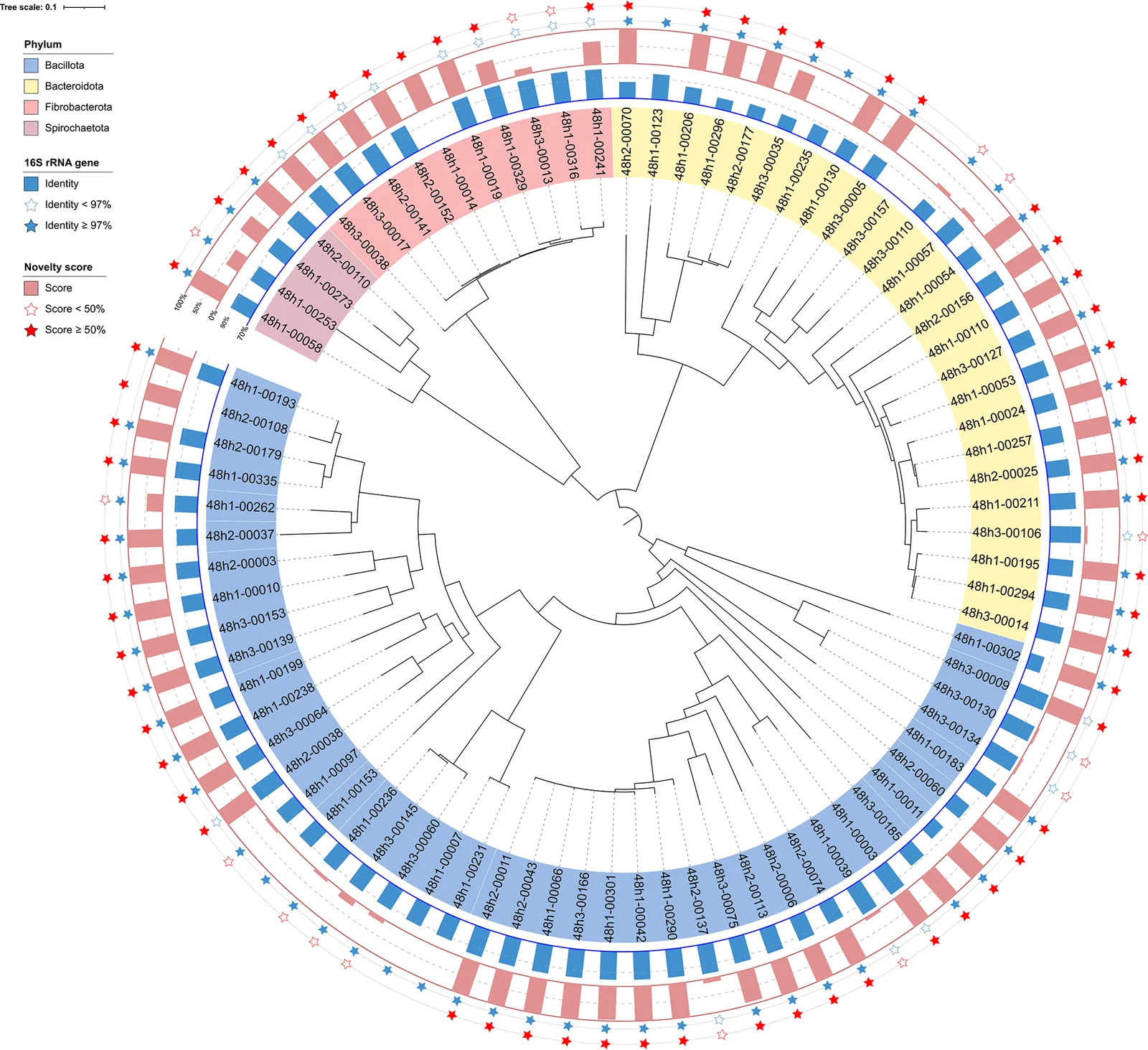
Phylogenetic tree of 82 bacterial single-cell amplified genomes (SAGs) collected from fiber-adhering rumen microorganisms. Archaeal SAG (48h3-00128) was removed to construct the phylogenetic tree based on bacterial single-copy core genes using bcgTree. The 16S rRNA genes were recovered from SAGs and searched using BLAST against the NCBI rRNA/ITS databases to calculate the identity with known species. The inner blue bar represents the calculated 16S rRNA gene identity. Three SAGs (48h1-00014, 48h2-00108, and 48h3-00157) did not contain 16S rRNA genes. The outer pink bar represents a genomic novelty score of SAGs calculated from average nucleotide identity and alignment fraction (Varghese *et al.*, 2015). Closed and open stars show greater than and less than 50% of the novelty score, respectively.

**Fig. 6.**
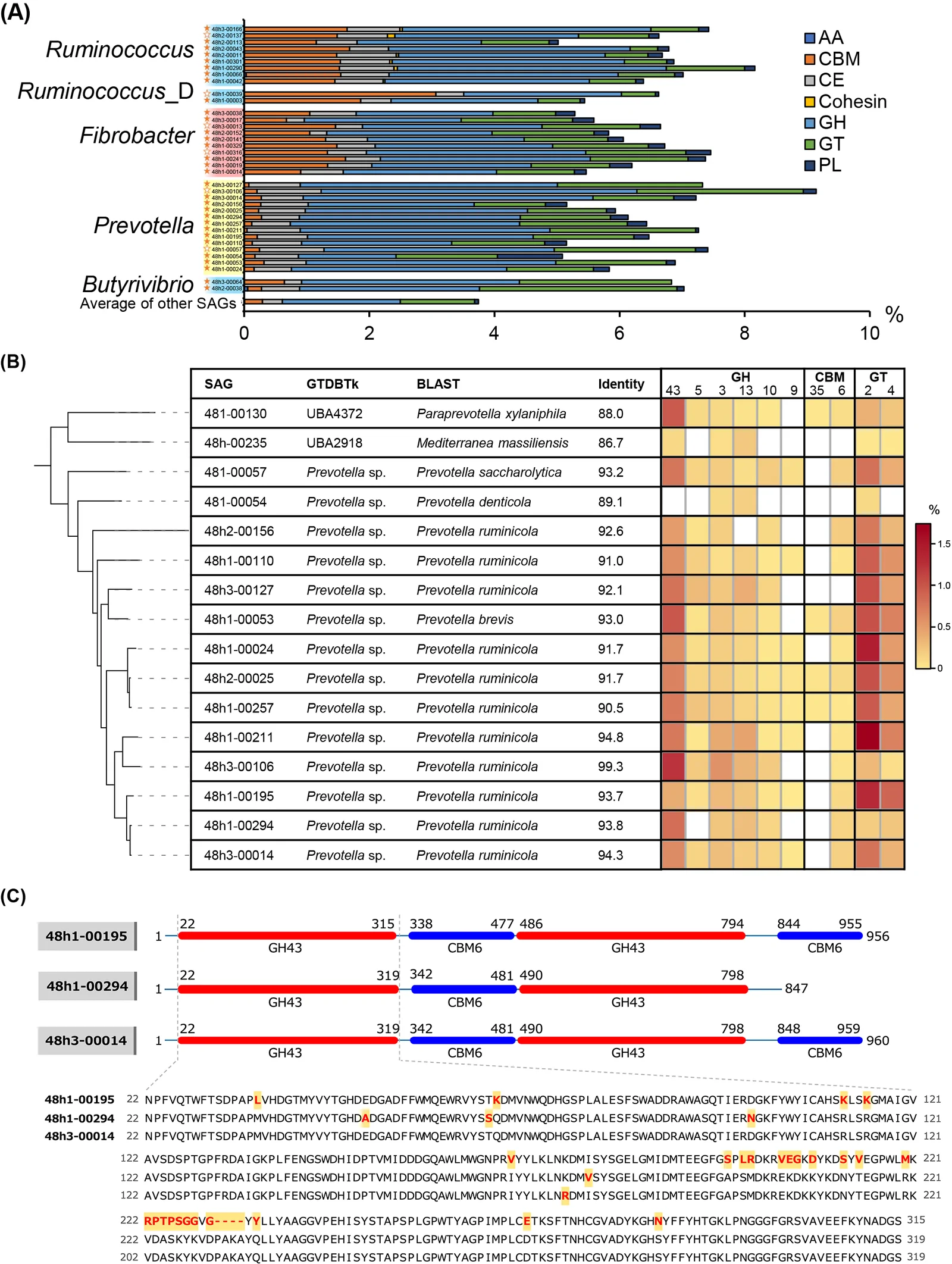
Resolution of a single-cell genomic anal­ysis. (A) Comparison of carbohydrate-active enzymes (CAZymes). SAGs of dominant taxa with a high percentage of CAZyme genes are shown individually, and other SAGs are included in “Average of other SAGs”. Closed and open stars show greater than and less than 50% of the novelty score, respectively. (B) A heatmap of major CAZymes and taxonomy of the dominant genus *Prevotella*. Numbers underlying CAZyme categories represent family classifications. The identity value represents a DNA sequence similarity of the 16S rRNA gene with the closest species. The dendrogram represents a phylogenetic tree based on bacterial single-copy core genes using bcgTree. (C) Polysaccharide utilization loci of three SAGs of uncultured *Prevotella* and the amino acid sequence of GH43. The positions representing a deletion or substitution are highlighted in yellow. AA, auxiliary activity; CBM, carbohydrate-binding module; CE, carbohydrate esterase; GH, glycoside hydrolase; GT, glycoside transferase; PL, polysaccharide lyase.

**Fig. 7.**
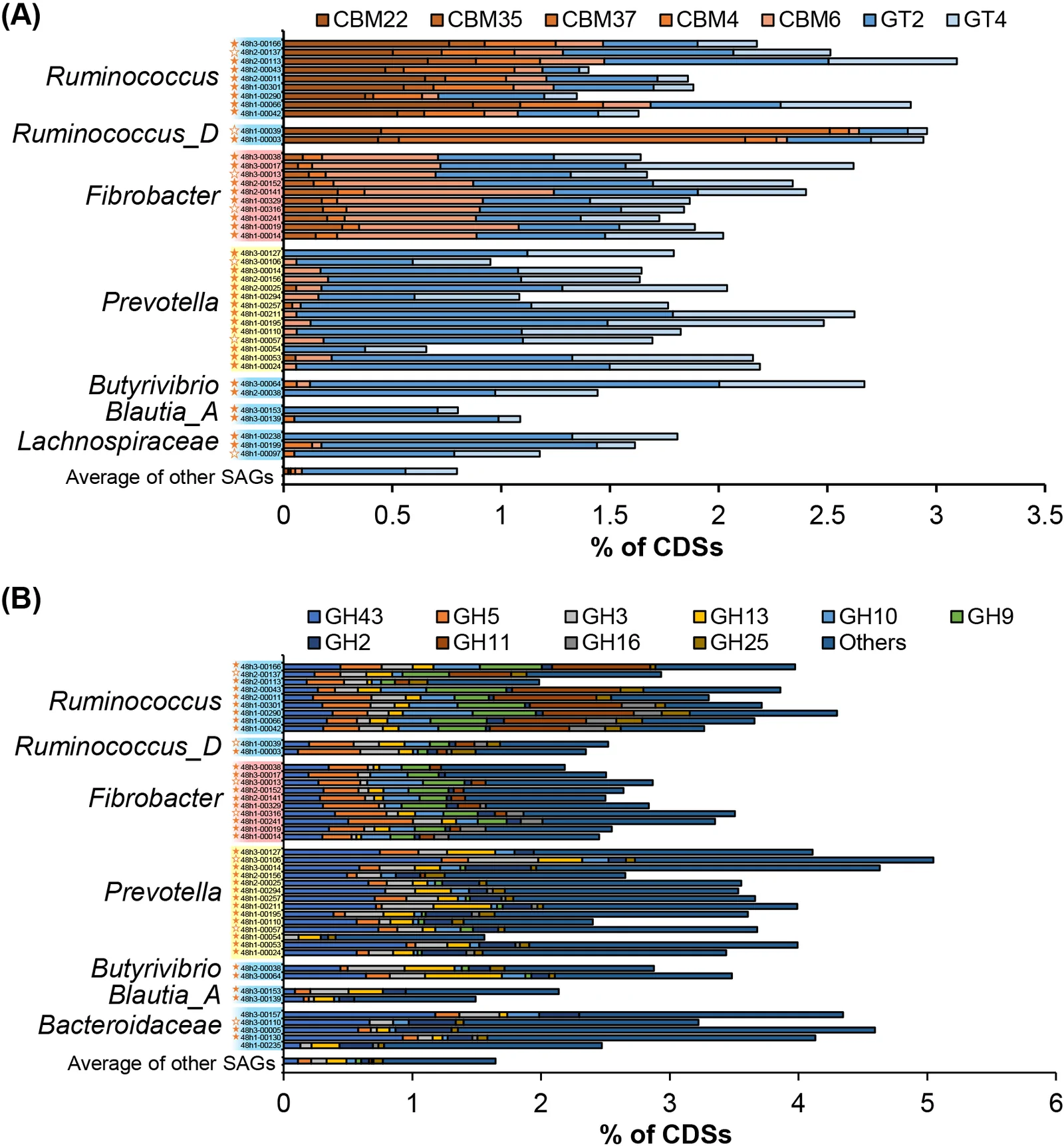
Profiles of single-cell amplified genomes (SAGs). (A) Carbohydrate-binding modules (CBM) and two glycoside transferase families (GT2 and GT4), which are involved in biofilm formation. The top 5 CBMs and 2 GTs involved in fiber adherence and biofilm formation are shown. (B) Glycoside hydrolases (GHs). The top 10 GHs are shown, and other GHs are included in “Others”. Closed and open stars show greater than and less than 50% of the novelty score, respectively.
