# Supplementary material for: Single-cell Genomic Analysis of Fiber-adhering Rumen Bacteria during an in situ Incubation of Timothy Hay
Source: Microbes Environ. 2026 Sep 29;41(3):ME26040. doi: 10.1264/jsme2.ME26040 (PMC13659345; doi:10.1264/jsme2.ME26040)
Supplement: Supplementary file 1 [file 41_26040_s1.pdf]

Supplementary Materials

Single-cell Genomic Analysis of Fiber-adhering Rumen Bacteria during an in situ Incubation of Timothy Hay

Shuhei Takizawa[1*], Miho Fujimori[1], and Takumi Shinkai[1]

[1] Institute of Livestock and Grassland Science, National Agriculture and Food Research Organization, 2 Ikenodai, Tsukuba, Ibaraki, Japan

*Corresponding author. E-mail: takizawa.shuhei011@naro.go.jp

Supplementary information:

Table S1. Quality of single-cell assembled genomes of fiber-adherent rumen microorganisms.

Table S2. Classification of single-cell assembled genomes of fiber-adherent rumen microorganisms.

Table S3. Profiles of functional genes involved in the fiber utilization of fiber-adherent rumen microorganisms.
